# Remote Clinics During Coronavirus Disease 2019: Lessons for a Sustainable Future

**DOI:** 10.7759/cureus.14114

**Published:** 2021-03-25

**Authors:** Alexander Curtis, Hammad Parwaiz, Courtney Winkworth, Lauren Sweeting, Laurence Pallant, Kaveh Davoudi, Eleanor Smith, Katherine Chin, Megan Kelsey, Andrew Stevenson

**Affiliations:** 1 Trauma and Orthopaedics, Musgrove Park Hospital, Taunton, GBR

**Keywords:** trauma & orthopedics, hospital sustainability, virtual clinic, t&o

## Abstract

Background

The coronavirus disease 2019 (COVID-19) pandemic has led to a focus on non-face-to-face (NF2F) orthopedic clinics. In this study, our aim was to establish whether NF2F clinics are sustainable according to the “triple bottom line” framework by taking into account the impact on patients, the planet, and the financial cost.

Methodology

This retrospective cohort study was carried out at a large district general hospital with 261 patients identified as having undergone face-to-face (F2F) or NF2F orthopedic consultations (April 2020). These patients were contacted by telephone to establish their experience, mode of transport, and preference for future consultations. Data were also collected relating to environmental and financial costs to the patient and the trust.

Results

The final analysis included 180 (69%) patients: 42% had an F2F consultation and 58% NF2F consultation. There was no significant difference between each group in terms of convenience, ease of communication, subjective patient safety, or overall satisfaction rating (p > 0.05). Overall, 80% of NF2F patients would be happy with virtual consultations in the future. The mean journey distance was 18.6 miles leading to a reduction in total carbon emissions of 563.9 kgCO_2_e (66%), equating to 2,106 miles in a medium-sized car. The hospital visit carbon cost (heating, lighting, and waste generation) was reduced by 3,967 kgCO_2_e (58%). The financial cost (petrol and parking) was also reduced by an average of £8.96 per person.

Conclusions

NF2F consultations are aligned to the National Health Service’s “Long Term Plan”: (i) delivering high patient satisfaction with equivalent outcomes as F2F consultations; (ii) reducing carbon emissions from transportation and hospital running; and (iii) becoming cheaper.

## Introduction

Novel coronavirus disease 2019 (COVID-19) was declared a pandemic by the World Health Organization [1]. It accounted for 26% of all deaths in the UK in March and April 2020, with a case-fatality rate of 15.3% [2]. Non-face-to-face clinics (NF2F) were encouraged to reduce the risk of transmission [3]. Prior to the pandemic, the use of virtual clinics formed a key component of the National Health Service’s (NHS) Long Term Plan [4], which aimed to reduce air pollution associated with travel. This goal is restated in “Delivering a Net Zero NHS,” which sets out the need for new sustainable models of care [5].

Within our trust, we reorganized our orthopedic emergency clinic to maximize NF2F appointments. This paper examines the sustainability of introducing such NF2F clinics. We used the triple bottom line (TBL) framework for this analysis by measuring the impact on patients, the planet, and the financial cost [6].

## Materials and methods

This retrospective cohort study was carried out at Musgrove Park Hospital, Taunton, UK which is a large district general hospital and trauma unit. All emergency orthopedic clinic referrals were screened by a consultant and allocated to either face-to-face (F2F) or NF2F clinic, depending on clinical factors. In total, 261 patients were identified from March 23, 2020 to April 30, 2020 using our electronic patient flow and appointments software (IMS MAXIMS, Milton Keynes, UK). These patients were contacted by telephone and asked questions about their experience, the mode of transport used to reach hospital, and their preferences for future consultations. A structured questionnaire was used with three attempts made to contact the patients. A total of 81 patients were excluded (Table 1), leaving 180 (69%) patients for analysis.

**Table 1:** Reasons for exclusion of patients from analysis. DNA, did not attend

Exclusion criteria	n
Inability to contact the patient by landline or mobile phone on three separate occasions	50
No contact number for the patient	14
Patient declined to answer questions	5
Currently an inpatient	4
Patient in a care home	3
DNA their appointment	3
Patient died	2
Total	81

Data were collected to quantify the effect of these clinics on patients, the environment, and the financial costs (Table 2).

**Table 2:** Data collected from each encounter. F2F, face-to-face; NF2F, non-face-to-face

Data item	
Patient demographics	Age and sex
Consultation type	F2F vs NF2F
Consultation satisfaction parameters	On a Likert scale (0-10)
Mode of transport	Car/bus/taxi/walked
Distance from hospital	Calculate CO_2_e
Preference for future consultation	F2F or NF2F (telephone/video)
Ability to use video consultation in future	Yes or no

Statistical analysis of quantitative data was performed using GraphPad Prism 8 software. Continuous data were analyzed using a two-tailed unpaired t-test following confirmation that the data were normally distributed.

## Results

Patient factors

In total, 76 (42%) patients had an F2F consultation and 104 (58%) had an NF2F consultation. The mean age was 48 (range: 3 months to 92 years), with 56% female and 44% male participants. Patient demographics did not vary significantly (p > 0.05) between groups.

There was no significant difference between the F2F and NF2F groups in terms of convenience (9.1/10 vs 9.2/10, p = 0.32), ease of communication (9.3/10 vs 9.2/10, p = 0.28), subjective patient safety (9.1/10 vs 9.2/10, p = 0.31), or overall satisfaction rating (9.0/10 vs 9.1/10, p = 0.35).

Overall, 80% of NF2F would be happy to be treated like this again in the future, and 33% of the F2F group would prefer to have an NF2F consultation in the future.

All NF2F consultations were telephone consultations. However, 84% reported that they had access and knowledge to perform a video consultation in the future. Those unable to use video consultation were significantly older by 17 years (p < 0.001).

Environmental factors

Overall, 82% of all patients either did (F2F) or would have (NF2F) attended the appointment using a car, 5% using a taxi, 3% using a bus, and 6% using another form of transport; only 4% walked (F2F) or would have walked (NF2F). The mean return journey distance from home to hospital was 18.6 miles. Estimates of the carbon dioxide emissions (CO_2_e) were made for each mode of transport (Table 3). In each transport group, there was a reduced CO_2_e of 65% for car, 84% for taxi, and 57% for bus journeys as a result of NF2F clinics. Overall, total carbon emissions were reduced by 563.9 kgCO_2_e (66%) or 3.1 kgCO_2_e per person. This equates to 2,106 miles driven in a medium-sized petrol car [7].

**Table 3:** Carbon emission according to the mode of transport for combined F2F and NF2F (assumed mode of transport) consultations. Data extracted from Gov.UK [7]. F2F, face-to-face; NF2F, non-face-to-face

Mode of transport	Carbon footprint (kgCO_2_e per mile)	Total carbon footprint (kgCO_2_e)
Medium-sized petrol car	0.26775	783.1
Taxi	0.26775	58.7
Bus	0.089 (per person)	9.0
Combined transport		850.8

In addition, there is an outpatient carbon cost associated with each visit (covering heating, lighting, and waste generated) of 56 kgCO_2_e per patient [8], and would have totaled 10,080 kgCO_2_e for all 180 patients. However, by utilizing NF2F consultations for 104 patients, this led to a 58% reduction of 5,846 kgCO_2_e.

Financial factors

The cost to the patient of attending an F2F consultation was also considered. This includes a cost of £4.40 ($6.00) for parking for two hours and average petrol cost of £4.56 ($6.22) for 82% of the patients who drove to their appointment. Those attending by bus paid £12.00 ($16.36) for the “day rider” ticket. Additionally, there may be a cost associated with missed work or organizing child care. We have not included the cost saving to the hospital of not having patients on site.

Table 4 illustrates the difference seen for an average individual patient who participated in an NF2F versus F2F consultation by car or bus when considering the TBL.

**Table 4:** Comparison of an average patient: (i) car F2F, (ii) bus F2F, (iii) NF2F in terms of carbon footprint from transport and consultation, financial cost to patient, and patient satisfaction. F2F, face-to-face; NF2F, non-face-to-face

Average patient	Transport carbon footprint (kgCO_2_e)	Consultation carbon footprint (kgCO_2_e)	Financial cost to patient (£)	Satisfaction (%)
Car F2F	5.3	38	8.96	89
Bus F2F	0.089	38	12.00	85
NF2F	0	0	0.00	91

## Discussion

There have been up to 70% fewer F2F fracture clinics in our trust since the start of the pandemic. We have embraced this change and will continue to offer NF2F clinic as often as possible. This is in line with the NHS’s “Long Term Plan” which seeks to cut one-third of all outpatient F2F consultations through digitalization [4].

Our study has shown that there is no significant difference in the convenience, safety, communication, and overall satisfaction rate of NF2F clinics versus F2F clinics. Most patients who had an NF2F clinic reported that they would be happy to have the same clinic appointment in the future. These positive findings are, in part, due to careful patient selection for NF2F appointments by a consultant. Broadly speaking, NF2F consultations were selected for patient groups (i) at low risk of a negative outcome, for example, a stable fracture unlikely to displace into an unsatisfactory position; (ii) not requiring a wound review; and (iii) with significant comorbidities that would put them at significant risk if they contract COVID-19. Our findings are very similar to Gilbert et al. [9] who found that their virtual orthopedic clinic had 90% satisfaction for both telephone and video consultations. This was multifactorial including: (i) reduced waiting times; (ii) reduced travel times; (iii) reduced impact on symptoms from traveling; and (iv) reduced risk of COVID-19 transmission. Clinicians also found consultations were more efficient. This efficiency was also noted by Hammersley et al. [10] who showed phone and video consultations to be 4.1 and 3.7 minutes shorter than F2F consultations, respectively.

We found that 84% of patients would have access to use video consultations, which can enable more intimate and personal consultation [11]. The experience of using video consultations is varied in the literature, with some technical difficulties experienced [9], while older patients were more likely to be excluded from this technology [9]. However, carefully selected patients can benefit from video consultations, especially when the patient and clinician already know and trust each other from previous consultations [11]. These consultations were more commonly selected by younger, more technologically experienced patients [10].

NF2F consultations reduce restrictions on patients who live in less accessible regions and provide greater patient choice on where they receive their treatment [12].

The financial cost to a patient attending an F2F consultation is significant, including the potential cost of missed work or organizing child care. These factors have been found to be important limitations for patients attending F2F consultations [13]. Certainly, studies have shown lower rates of non-attendance in NF2F groups versus F2F groups [14], which is important given that 9% of all hospitals appointments are not attended, costing the NHS £225 million per year ($307 million per year) [15].

Given the current financial deficit of the NHS [16], any opportunity to sustainably reduce costs is important. Studies have shown that NF2F surgical consultations can lead to savings of up to 89%, with attendance increasing by 7.1% [17].

The Earth is in a state of climate and ecological emergency. If greenhouse gas emissions continue at current levels the world will heat up by 4 degrees by 2100 [18]. These emissions will not only heat the earth but also cause significant harm to health through atmospheric pollution. Air pollution, particularly around hospital sites caring for vulnerable individuals, is a major risk factor. There are 248 hospitals and 2,000 health centers in Great Britain which exceed the safe air pollution level [19]. Air pollution is linked to 36,000 deaths in England annually [20].

NHS England is committed to becoming Net Zero in terms of carbon emissions by 2040 [5]. In England, 3.5% of all road travel is related to the NHS with 6.7 billion road miles each year due to patients and visitors traveling to NHS facilities [20].

The World Health Organization has set the acceptable limit for fine particular matter pollution at 10 μg/m^3^ [21]. Our hospital (Musgrove Park Hospital, Taunton) is currently at the upper end of the acceptable limit with a pollution level of 8.62 μg/m^3^. Reduction in vehicle travel would help to reduce this.

Limitations of this study include patient exclusion bias with 19% of patients not contactable despite three attempts to contact them. It is possible that the patients who were not contactable may also have had more positive or negative perceptions of NF2F consultations. Additionally, this study was conducted at the height of the early phase of the pandemic and may have impacted patient experiences and answers to the questions. We recommend re-evaluation of patient preferences once the prevalence of COVID-19 recedes.

## Conclusions

This study shows that NF2F consultations are acceptable to patients in terms of satisfaction and cost and reduce CO_2_e when considering the transport and running costs of a clinic. We would advocate the use of NF2F clinics, where clinically appropriate, as a sustainable model of care.

## References

[1] (2020). World Health Organization coronavirus disease 2019 (COVID-19) situation report-51. https://www.who.int/docs/default-source/coronaviruse/situation-reports/20200311-sitrep-51-covid-19.pdf.

[2] (2020). John Hopkins University & Medicine: mortality analyses. https://coronavirus.jhu.edu/data/mortality.

[3] (2020). NHSx: COVID-19 Information Governance advice for staff working in health and care organisations. https://www.nhsx.nhs.uk/covid-19-response/data-and-information-governance/information-governance/covid-19-information-governance-advice-health-and-care-professionals/.

[4] (2020). National Health Service: The NHS long term plan. https://www.longtermplan.nhs.uk/.

[5] (2020). NHS: delivering a ‘Net Zero’ National Health Service. https://www.england.nhs.uk/greenernhs/wp-content/uploads/sites/51/2020/10/delivering-a-net-zero-national-health-service.pdf.

[6] (2020). The Economist: triple bottom line. https://www.economist.com/news/2009/11/17/triple-bottom-line.

[7] (2020). GOV.UK: greenhouse gas reporting: conversion factors 2020. https://www.gov.uk/government/publications/greenhouse-gas-reporting-conversion-factors-2020.

[8] (2020). NHS Sustainable Development Unit: good and services carbon hotspots. https://www.sduhealth.org.uk/policy-strategy/reporting/natural-resource-footprint-2018/carbon-hotspots.aspx.

[9] Gilbert AW, Billany JCT, Adam R (2020). Rapid implementation of virtual clinics due to COVID-19: report and early evaluation of a quality improvement initiative. BMJ Open Qual.

[10] Hammersley V, Donaghy E, Parker R (2019). Comparing the content and quality of video, telephone, and face-to-face consultations: a non-randomised, quasi-experimental, exploratory study in UK primary care. Br J Gen Pract.

[11] Greenhalgh T, Shaw S, Wherton J (2018). Real-world implementation of video outpatient consultations at macro, meso, and micro levels: mixed-method study. J Med Internet Res.

[12] Harrison R, Macfarlane A, Murray E, Wallace P (2006). Patients' perceptions of joint teleconsultations: a qualitative evaluation. Health Expect.

[13] Car J, Sheikh A (2003). Telephone consultations. BMJ.

[14] Salisbury C, Foster NE, Hopper C (2013). A pragmatic randomised controlled trial of the effectiveness and cost-effectiveness of 'PhysioDirect' telephone assessment and advice services for physiotherapy. Health Technol Assess.

[15] (2020). National Audit Office: NHS waiting times for elective care in England. https://www.nao.org.uk/report/nhs-waiting-times-elective-care-england-2/.

[16] (2020). National Audit Office: NHS financial sustainability. https://www.nao.org.uk/report/nhs-financial-sustainability/.

[17] Fischer K, Hogan V, Jager A, von Allmen D (2015). Efficacy and utility of phone call follow-up after pediatric general surgery versus traditional clinic follow-up. Perm J.

[18] Intergovernmental Panel on Climate Change (2020). Intergovernmental Panel on Climate Change: A report of Working Group I of the Intergovernmental Panel on Climate Change. https://www.ipcc.ch/site/assets/uploads/2018/02/ar4-wg1-spm-1.pdf.

[19] (2020). British Lung Foundation: toxic air at the door of the NHS. https://www.blf.org.uk/take-action/campaign/nhs-toxic-air-report.

[20] (2020). National Health Service: greener NHS campaign to tackle climate ‘health emergency’. https://www.england.nhs.uk/2020/01/greener-nhs-campaign-to-tackle-climate-health-emergency/.

[21] (2020). World Health Organization: ambient (outdoor) air quality and health: fact sheet. https://web.archive.org/web/20160104165807/http://www.who.int/mediacentre/factsheets/fs313/en/.

